# GPC3 Promotes Lung Squamous Cell Carcinoma Progression and HLA-A2-Restricted GPC3 Antigenic Peptide-Modified Dendritic Cell-Induced Cytotoxic T Lymphocytes to Kill Lung Squamous Cell Carcinoma Cells

**DOI:** 10.1155/2023/5532617

**Published:** 2023-11-06

**Authors:** Jing Ning, Jianqiao Ding, Shu Wang, Youhong Jiang, Daqing Wang, Shenyi Jiang

**Affiliations:** ^1^Department of General Medicine, Cancer Hospital of Dalian University of Technology, Cancer Hospital of China Medical University, Liaoning Cancer Hospital & Institute, Shenyang 110042, China; ^2^Molecular Oncology Department of Cancer Research Institution, The First Affiliated Hospital of China Medical University, Shenyang 110001, China; ^3^Department of Thoracic Surgery (2), Cancer Hospital of Dalian University of Technology, Cancer Hospital of China Medical University, Liaoning Cancer Hospital & Institute, Shenyang 110042, China; ^4^Department of Radiotherapy, The Second Hospital of Jilin University, Changchun 130000, China; ^5^Hope Plaza Children's Hospital District of Dalian Municipal Women and Children's Medical Center, Dalian 116000, China; ^6^Department of General Practice, The First Affiliated Hospital of China Medical University, Shengyang 110001, China

## Abstract

Lung squamous cell carcinoma (LUSC) is associated with poor clinical prognosis and lacks available targeted agents. GPC3 is upregulated in LUSC. Our study aimed to explore the roles of GPC3 in LUSC and the antitumor effects of HLA-A2-restricted GPC3 antigenic peptide-sensitized dendritic cell (DC)-induced cytotoxic T lymphocytes (CTLs) on LUSC. LUSC cells with GPC3 knockdown and overexpression were built using lentivirus packaging, and cell viability, clone formation, apoptosis, cycle, migration, and invasion were determined. Western blotting was used to detect the expression of cell cycle-related proteins and PI3K-AKT pathway-associated proteins. Subsequently, HLA-A2-restricted GPC3 antigenic peptides were predicted and synthesized by bioinformatic databases, and DCs were induced and cultured *in vitro*. Finally, HLA-A2-restricted GPC3 antigenic peptide-modified DCs were co-cultured with T cells to generate specific CTLs, and the killing effects of different CTLs on LUSC cells were studied. A series of cell function experiments showed that GPC3 overexpression promoted the proliferation, migration, and invasion of LUSC cells, inhibited their apoptosis, increased the number of cells in S phase, and reduced the cells in G2/M phase. GPC3 knockdown downregulated cyclin A, c-Myc, and PI3K, upregulated E2F1, and decreased the pAKT/AKT level. Three HLA-A2-restricted GPC3 antigenic peptides were synthesized, with GPC3_522-530_ FLAELAYDL and GPC3_102-110_ FLIIQNAAV antigenic peptide-modified DCs inducing CTL production, and exhibiting strong targeted killing ability in LUSC cells at 80 : 1 multiplicity of infection. GPC3 may advance the onset and progression of LUSC, and GPC3_522-530_ FLAELAYDL and GPC3_102-110_ FLIIQNAAV antigenic peptide-loaded DC-induced CTLs have a superior killing ability against LUSC cells.

## 1. Introduction

Lung cancer is one of the most harmful cancers worldwide owing to its high incidence (2.206 million cases, 11.4%) and high mortality (1.796 million cases, 18%), which seriously threatens human health [1]. The two main pathological types of lung cancer are small cell lung cancer (SCLC) and non-small cell lung cancer (NSCLC); NSCLC accounts for about 85% of lung cancer [2]. In NSCLC, lung squamous cell carcinoma (LUSC) has poor treatment sensitivity and prognosis; it is commonly characterized by central type occupying space and is highly prevalent in the smoking population [3]. The primary therapeutic methods for lung cancer include surgery, chemotherapy, radiotherapy, targeted drug therapy, and immunotherapy [4]. Immunotherapy includes active immunotherapy and passive immunotherapy; the most commonly used method in clinical practice involves immune checkpoint inhibitors (ICIs), which belong to active immunotherapy. Approximately 30% of patients can benefit from the application of ICIs in clinical practice [5]. Recently, an increasing number of targeted therapies are approved for the treatment of NSCLC [6, 7]. However, genotype-matched targeted therapy that responds to LUSC patients has not been elucidated [8]. Therefore, it is warranted to discover potential therapeutic targets and novel immunotherapy for LUSC to improve therapeutic efficacy.

The tumor microenvironment (TME) carries the complex mechanism of the human immune system against the tumor and plays an important role in tumor development [9]. Tumor infiltration lymphocyte (TIL)-mediated antitumor immune response takes cellular immunity as the core and especially cytotoxic T lymphocytes (CTLs) that are the main effector cells of antitumor immunity [10]. The key to antitumor immune response is that antigen presenting cells (APCs) present tumor-associated antigens to CTLs to activate the cellular immune system of the body to eliminate tumor cells [11]. The presentation of tumor-associated antigens must be degraded to short peptides by APCs, and then bound to major histocompatibility complex (MHC) molecules to form peptide-MHC-TCR complexes for recognition by T cells that stimulate the response of CTLs [12, 13]. The effective binding of human leukocyte antigen (HLA) and tumor antigen is the beginning of immunotherapy; antitumor immunity by antigen-specific T cells depends on good binding to HLA and the epitope [14]. Therefore, accurate HLA typing is a prerequisite for immunotherapy based on individual tumor epitopes, and the determination of effective molecular targets is the key to immunotherapy. HLA-A2 is a type of HLA, and HLA-A2-restricted tumor antigen peptide is identified as a novel vaccine for cancer immunotherapy [15]. A HLA-A2-restricted CTL epitope induces antitumor effects against human lung cancer in mouse xenograft model [16]. Another study identified two myeloperoxidase-derived HLA-A2-restricted peptides (MY4 and MY8) that are immunogenic, and MY4- and MY8-specific cytotoxic T lymphocytes may play a role in reducing leukemia; this indicates that MY4 and MY8 may be novel leukemia-associated antigens for immunotherapy in myeloid leukemia [17]. Tumor infiltration lymphocytes kill tumors and mediate changes to the immunosuppressive TME; this leads to the weakening of antitumor immunogenicity, so that tumor cells can implement immune escape [18]. Therefore, improving tumor immunogenicity and regulating TILs in TME to improve the autoimmune response may be effective ways to fight against tumors.

Dendritic cells (DCs) are the most important professional APCs that take in, process, and present antigens *in vivo*; they have the strongest ability to present antigens [19]. The antigen recognition ability of the tumor-bearing body decreases owing to the change of TME, and DCs do not fully play the role of antigen presentation owing to the lack of effective load of specific antigen; this results in DC dysfunction and leads to the decreased immunogenicity of tumor cells [20]. In recent years, modified DCs can enhance the immunogenicity of tumors and specifically target to kill tumor cells by effectively recognizing activated CTLs [21, 22]. Although researchers have made great progress in understanding the role and function of DCs, the targeting of specific antigen-loaded DCs to improve antitumor immunity remains to be investigated.

Glypican-3 (GPC3) is a heparan sulfate proteoglycan (HSPG) anchored to the cell membrane that participates in a variety of biological processes and different cell signaling pathways through binding with receptor ligands, such as growth factors, cell adhesion molecules, matrix components, and so on, thereby playing a crucial role in embryonic development, tumorigenesis, and progression [23, 24]. GPC3 is generally overexpressed in tumor tissues, including lung cancer, but is almost not expressed in the corresponding normal tissues [25–27]. The percentage of samples with predicted GPC3 overexpression was 45% for LUSC and 8% for adenocarcinoma of the lung (LUAD) based on the functional genomic mRNA profiling of a large cancer database [25]. GPC3 protein expression is significantly higher in LUSC than in LUAD [26]. This indicates that GPC3 may be a candidate marker to detect LUSC. There are many tumor antigens in LUSC, including MAGE-A3, MAGE-A4, and NE-ESO-1 [28]. Besides, CD8+ effector cells can infiltrate into LUSC more extensively than other adenocarcinomas [29]. Genomic analysis of LUSC found that many samples show mutational inactivation of the HLA-A class I major histocompatibility genes that may contribute to the loss of immune function and downregulation of tumor antigens [30]. Additionally, GPC3 (144–152) peptide vaccine can induce high avidity CTLs capable of killing hepatocellular carcinoma (HCC) cells expressing GPC3 that may be used for HCC immunotherapy [31]. Therefore, the near-zero expression of GPC3 in normal tissues, low expression in LUAD, and high expression in LUSC led us to hypothesize that GPC3 plays a role in immunotherapy as a tumor-associated antigen of LUSC and becomes a reasonable immunotherapeutic target for LUSC.

Our previous bioinformatic analysis found that GPC3 was highly expressed in the LUSC tissues compared to the paracarcinoma tissues and was involved in LUSC cells proliferation [32]. This study initially explored the effects and mechanisms of GPC3 in LUSC malignant biological behavior at the cellular level, and then investigated the antitumor effects of HLA-A2-restricted GPC3 antigenic peptide-sensitized DC-induced CTLs on LUSC. The outcomes of this study may help further illustrate the roles of GPC3 in LUSC and provide a theoretical basis to discover new targets of LUSC immunotherapy.

## 2. Materials and Methods

### 2.1. Data Download and Bioinformatics Analysis

The RNA-seq expression data of LUSC were downloaded from The Cancer Genome Atlas (TCGA) database (https://www.cancer.gov/ccg/research/genome-sequencing/tcga), which contained 501 LUSD samples. Then, Tumor Immune Dysfunction and Exclusion (TIDE) database (http://tide.dfci.harvard.edu/) was used to elevate the response of each LUSD sample to immune checkpoint therapy as a TIDE score. Kruskal–Wallis test was used to compare the distribution difference of *GPC3* expression between the different response types (non-responder and responder). At the same time, other indicators of immunotherapy were also obtained, including TIDE score, interferon-*γ* (IFN-*γ*), microsatellite instability (MSI), Merck18, cancer-associated fibroblasts (CAF), and Cor function were used to calculate the correlation between each indicator and *GPC3* expression level. Additionally, the expression levels of immune checkpoint genes (*CD27*, *CD40*, *CD70*, *CD86*, *CTLA4*, *HAVCR2*, *ICOS*, *IDO1*, *PDCD1*, *PDL1*, *TIGIT*) were extracted from LUSD data in TCGA, and then the correlation between the expression levels of each immune checkpoint gene and GPC3 expression levels was calculated.

### 2.2. Cell Culture

Human LUSC cell lines (NCI-H226 and SK-MES-1) and a human normal alveolar epithelial cell line (BEAS-2B) were, respectively, obtained from the National Collection of Authenticated Cell cultures (Shanghai, China) and BeNa Culture Collection (Beijing, China). Among them, LUSC cell lines were cultured in RPMI-1640 medium (Servicebio, Wuhan, China) with 10% fetal bovine serum (FBS) (Servicebio), while BEAS-2B cells were cultured in Dulbecco's Modified Eagle's Medium (DMEM) (Servicebio) supplemented with 10% FBS (Servicebio). They were all maintained in an incubator with 5% CO_2_ at 37°C. Cell lines were passaged after reaching 80%–90% confluence.

### 2.3. Acquisition of shGPC3/oeGPC3 LUSC Stable Transfection Cell Lines

The shRNA GPC3 vectors (shGP3-1, shGPC3-2, and shGPC3-3) and overexpressed GPC3 vectors (GPC3-OE) were purchased from Shanghai Genechem Co., Ltd. (Shanghai, China). The cell transfection methods were performed as previously reported [33]. Briefly, NCI-H226/SK-MES-1 cells were seeded into a 24-well plate at a density of 1 × 10^5^ cells/well and cultured overnight. The medium was changed to serum-free medium, and the cells were transfected with either shGPC3-control, shGP3-1, shGPC3-2, shGPC3-3, GPC3-OE-control, or GPC3-OE for 12 hr using Lipofectamine 3000 (Thermo Fisher Scientific, Waltham, MA, USA) following the manufacturer's instructions. The medium was replaced with complete medium, and the cells were cultured for another 48 hr. The medium was changed to complete medium supplemented with puromycin (2 *μ*g/mL) to select resistant cells. Finally, the shGPC3-NCI-H226/SK-MES-1 or oeGPC3-NCI-H226/SK-MES-1 stable transfection cell lines were chosen and expanded in culture for subsequent experiments. Total RNA was extracted from the cells with different treatments, and the cell transfection efficiency was evaluated by measuring *GPC3* expression using quantitative real-time polymerase chain reaction (RT-qPCR) and western blotting.

### 2.4. RT-qPCR

Total RNA was isolated from NCI-H226 cells, SK-MES-1 cells, and BEAS-2B cells or cells with different treatments using RNAiso Plus (Trizol, Takara) following the manufacturer's protocols. The purity and concentration of total RNA were determined using a microplate reader. Then, the isolated RNA was reverse-transcribed into cDNA using a PrimeScript™ RT Reagent Kit with gDNA Eraser (Takara) following the manufacturer's recommendations. The primer sequences for amplifying *GPC3* and *GAPDH* were: *GPC3*: F, 5′ CCTGGATGAGGAAGGGTTTG 3′; R, 5′ GGAGTTGCCTGCTGACTGTTT 3′; *GAPDH*, F, 5′ CAGGAGGCATTGCTGATGAT 3′; R, 5′ GAAGGCTGGGGCTCATTT 3′. *GAPDH* was used as a housekeeping gene. TB Green® Premix Ex Taq™ II (Tli RNaseH Plus) was used for qPCR, and the RT-qPCR reaction was initiated at 95°C for 30 s, followed by 40 cycles of 95°C for 5 s and 60°C for 30 s. The relative mRNA expression of *GPC3* was calculated using the 2^−*ΔΔ*Ct^ method.

### 2.5. Western Blotting

Total protein was extracted from NCI-H226 cells, SK-MES-1 cells, and BEAS-2B cells or the cells with different treatments using RIPA lysis buffer (Beyotime Biotechnology, Shanghai, China). A BCA Protein Assay Kit (Beyotime Biotechnology) was utilized to examine the total protein concentrations. The protein samples (20 *μ*g) were separated by 10% sodium dodecyl sulfate-polyacrylamide gel electrophoresis (SDS-PAGE) and transferred to polyvinylidene difluoride (PVDF) membranes. After blocked with 5% skim milk at 37°C for 1.5 hr, the membranes were incubated overnight at 4°C with anti-GPC3 antibody (1 : 5,000, Abcam), anticyclin A antibody (1 : 10,000, Proteintech), anti-c-Myc antibody (1 : 10,000, Proteintech), anti-E2F1 antibody (1 : 4,000, Proteintech), anti-PI3K antibody (1 : 15,000, Proteintech), anti-AKT antibody (1 : 10,000, Proteintech), anti-pAKT antibody (1 : 5,000, Proteintech), or anti-GAPDH antibody (1 : 8,000, Proteintech). The membranes were incubated with secondary antibody (1 : 10,000, Proteintech) for 1 hr at 37°C. After washing, the protein bands were visualized using a ECL Chemiluminescence Kit and Millipore ECL System (EMD Millipore Inc., Massachusetts, USA).

### 2.6. Immunofluorescence (IF) Assay

The NCI-H226 cells and SK-MES-1 cells with GPC3 knockdown or overexpression were seeded into a 96-well plate at a density of 7,500 cells/well and grown to a confluence of 90%. The medium was removed and washed with phosphate-buffered saline (PBS). The cells were fixed with 4% paraformaldehyde and sealed with dyeing sealing fluid (Beyotime Biotechnology) in the dark for 2 hr. The cells were washed, incubated with anti-GPC3 antibody overnight at 4°C, and then incubated with fluorescent secondary antibody for 1 hr at 37°C in the dark. The cells were washed, sealed with 4′,6-diamidino-2-phenylindole (DAPI) at room temperature for 10 min in the dark, and then sealed with neutral resins containing antifluorescent quenching agent. Finally, the images were observed and acquired under a laser confocal microscope (Nikon A1R, Japan).

### 2.7. Cell Viability and Clone Formation Assays

The differently treated cells were harvested, and Cell Counting Kit-8 (CCK-8, Beyotime Biotechnology) was used to determine cell viability. Briefly, the cells were seeded into a 96-well plate, cultured for 0, 24, 48, and 72 hr, followed by the addition of 10 *μ*L CCK-8 reagent to each well. The plate was incubated for 4 hr at 37°C, a microplate reader was used to measure the absorbance at 450 nm, and the cell viability curves were drawn.

The differently treated cells were cultured in an incubator for approximately 2–3 weeks for cell clone formation. The cells were continuously observed during incubation, and the culture was terminated when obvious cell clones were visible in the dish. The supernatant was removed, the cells were washed three times with PBS, and fixed with 2 mL methyl alcohol at room temperature for 15 min. The fixative was discarded, and the cells were stained with crystal violet (2 mL) for 30 min. The cells were washed three times with PBS, dried, and the cell colonies were photographed and counted.

### 2.8. Cell Apoptosis and Cell Cycle Assays

The Annexin V-FITC/PI Double Staining Assay Kit (Beyotime Biotechnology) was used to determine the apoptosis of the cells according to the manufacturer's instructions. Briefly, the cells were collected by centrifugation at 1,000×*g* for 5 min, resuspended in 195 *μ*L Annexin V-FITC binding buffer, followed by the addition of 5 *μ*L Annexin V-FITC staining solution and 10 *μ*L PI (50 *μ*L/mL). The treated cells were incubated for 20 min at 25°C in the dark, and the cells were placed in an ice bath. During incubation, the cells were resuspended three times to improve the staining effect. Annexin V-FITC showed green fluorescence, and PI exhibited red fluorescence. Finally, a flow cytometer (Becton, Dickinson and Company, USA) was utilized to detect the cells, and the apoptosis rate was calculated using FlowJo 10.4 software.

The cell cycle assay used a PI single dyeing method and a flow cytometer. The cells were resuspended in 1 mL precooled PBS, fixed with precooled 70% ethanol (1 mL) overnight at 4°C, centrifuged at 1,000×*g* for 5 min, and the sediments were resuspended with 1 mL precooled PBS. The resuspension was centrifuged, followed by the addition of 0.5 mL PI staining solution to stain the cells, and the mixture was incubated for 30 min at 37°C. Ultimately, the red fluorescence was detected by flow cytometry at an excitation wavelength of 488 nm, and the light scattering was detected. The cell cycle was analyzed using FlowJo 10.4 software.

### 2.9. Cell Migration and Invasion Assays

The scratch test was used to determine the migration of the cells with different treatments. The shGPC3-NCI-H226/SK-MES-1 cells or oeGPC3-NCI-H226/SK-MES-1 cells were seeded into a six-well plate at a confluence of 80%. Cells were cultured to 100% confluence, and then a micropipettor with a measuring range of 10 *µ*L was used to gently draw one horizontal line and one vertical line along the central axis of the six-well plate. The cells were washed twice with PBS, followed by the addition of complete medium, and incubation with 5% CO_2_ at 37°C. The migration distance of the cells was observed by taking pictures under a positive microscope at 0, 12, and 24 hr in the culture process.

Additionally, cell migration and invasion were assessed using Transwell chambers (pore size 5 *μ*m; Corning Inc., USA). Cell invasion assays involved initially coating the upper Transwell chambers with 100 *μ*L Matrigel Matrix (Corning Inc., USA, Matrigel Matrix: PBS = 1 : 8). The cells were harvested and inoculated at the upper chamber of the Transwell at the density of 5 × 10^4^ cells. The lower chamber of the Transwell was the complete medium with 20% FBS. The cells were cultured for 24 h, washed with PBS, fixed with 4% paraformaldehyde for 30 min, washed, stained with 500 *μ*L crystal violet for 30 min, washed, and dried. The cell images were observed under a microscope, and 10 fields were randomly selected. The positive cells were stained purple, and the average cell number was calculated.

### 2.10. Patient Collection and Primary LUSC Cell Culture

A total of six patients who underwent lobectomy owing to “pulmonary mass” were recruited in Liaoning Cancer Hospital. Blood samples of the six patients were collected before operation and confirmed to be HLA-A2 positive. The excised lung mass tissues of the patients (about 1 × 1 × 0.5 cm) were taken as the experimental group, and their corresponding peripheral lung tissues more than 10 cm away from the masses were taken as the normal control group. The obtained tissues were stored in RPMI-1640 medium containing 1% penicillin/streptomycin. All the experiments were performed in accordance with relevant guidelines and regulations (such as the Declaration of Helsinki) and approved by the Ethics Committee of Liaoning Cancer Hospital (approval no. 20210102K). Informed consent was obtained from all the subjects.

The obtained tissues were washed with PBS, cut into pieces, and digested with 5 mL 0.1% type I collagenase digestion solution containing 1% penicillin/streptomycin for 60 min at 37°C. Subsequently, 5 mL complete medium was added to terminate the digestion. The sediments were filtered through an 80-mesh screen, centrifuged at 800×*g* for 5 min at 4°C, resuspended with complete medium, and cultured for 12 hr at 37°C in an incubator with 5% CO_2_. The original medium was replaced with fresh medium when cell adhesion was observed. The cells were subcultured and frozen in appropriate proportions according to the growth state of the cells.

### 2.11. Prediction, Design, Synthesis, and Purification of HLA-A2-Restricted GPC3 Antigenic Peptides

HLA-A2-restricted GPC3 antigenic peptides were predicted, designed, synthesized, and purified by Sangon Biotech Co., Ltd. (Shanghai, China). Briefly, the full-length amino acid sequence of GPC3 (Isoform1, identifier: P51654-1) was obtained based on the Uniprot database (https://www.uniprot.org/uniprot/P51654), and online databases such as NetMHC 4.0 (http://www.cbs.dtu.dk/services/NetMHC), SYFPEITHI (http://www.syfpeithi.de/bin/MHCServer.dll/EpitopePrediction.htm), IEDB (http://www.iedb.org/), and Propred-1 (https://webs.iiitd.edu.in/raghava/propred1/index.html) were used to predict HLA-A2-restricted GPC3 epitopes. Peptides were designed according to a concentrated epitope region, and were synthesized based on Fmoc solid-phase principle using the polypeptide synthesizer [34]. High-performance liquid chromatography (HPLC) was used to purify the crude peptides obtained after synthesis, and the purified peptides were identified and analyzed on an API 2000 mass spectrometer. The obtained peptide was the target peptide when the measured molecular weight was consistent with the theoretical value. The identified peptide solution was lyophilized, dissolved in dimethyl sulfoxide (DMSO), and stored at −20°C.

### 2.12. Induction and Culture of DCs In Vitro

Two HLA-A2 female volunteers were selected and signed informed consent that was approved by the Ethics Committee of Liaoning Cancer Hospital (approval no. 20210102K). HLA subtypes in the peripheral blood of healthy volunteers were detected by flow cytometry. The methods of DC induction and culture are described as follows: 20 mL peripheral blood of the two healthy volunteers were collected, transferred to 50-mL centrifugal tubes, and centrifuged at 2,000 rpm for 9 min. The supernatant was transferred to a new 15-mL tube, heated at 56°C for 30 min, and stored at 4°C for use. The plasma samples were diluted with an equal volume of PBS and slowly added to the upper layer of lymphocyte separation solution (Tianjin Haoyang Biological Products Technology Co., Ltd., Tianjin, China). The sample was centrifuged at 2,000 rpm for 18 min, and the white membrane layer was transferred to a new 15-mL centrifuge tube. The cells were washed twice with PBS and centrifuged at 2,000 rpm for 8 min. The sediments were resuspended in 10 mL RPMI-1640 medium, transferred to a 75 cm^2^ culture flask, and incubated at 37°C for 2 hr with 5% CO_2_. DCs were adherent, while the nonadherent cells in the supernatant were transferred to a new culture flask, supplemented with medium, and cultured. The DCs were added with 100-mL RPMI-1640 medium containing 5% autologous plasma, 500 U/mL interleukin-4 (IL-4) (Sigma-Aldrich), and 1,000 U/mL granulocyte-macrophage colony-stimulating factor (GM-CSF) (Thermo Fisher Scientific) and incubated at 37°C. The DCs were observed, and the medium was changed at half volume every 3 days and supplemented with cytokines. The cells were divided into the control group and experimental group on the 5th day. The cells in the experimental group were supplemented with 50 mg/mL peptides, while the cells in the control group were untreated. The cells in each group were treated with 1,000 U/mL tumor necrosis factor-*α* (TNF-*α*) and incubated. Mature DCs were harvested on the 7th day of culture.

### 2.13. Detection of HLA-A2 and DC-Related Marker (CD11c, HLA-DR, CD80, CD86, CD83) Expression

Flow cytometry was used to detect the expression of HLA-A2 in peripheral blood of healthy volunteers, and the expression of DC-related markers in immature DCs on the 3rd day, the DCs in the control group on the 7th day, and the DCs in the experimental group on the 7th day. Meanwhile, the peripheral blood samples were centrifuged at 1,000 rpm for 5 min, and 3 mL red blood cell lysis buffer was added. The sample was incubated on ice for 5 min, and then 10 mL cell staining buffer was added to terminate cell lysis. The sample was centrifuged at 1,000 rpm for 5 min, and the sediments were resuspended at a density of 10^6^ cells/tube. The obtained DCs following different treatments were collected by centrifugation at 1,200 rpm for 5 min. The cells were washed twice with PBS, and then resuspended in PBS at 10^6^ cells/tube. After that, the processed samples were added with HLA-A2-FITC antibody (BioLegend), CD11c-FITC antibody (BioLegend), HLA-DR-FITC antibody (BioLegend), CD80-PE antibody (BioLegend), CD86-APC antibody (BioLegend), CD83-PE antibody (BioLegend), and their corresponding isotype control antibody. The cells were incubated for 40 min at 4°C in the dark, washed twice with PBS, resuspended with 500 *μ*L PBS, and analyzed by a flow cytometry.

### 2.14. Enzyme-Linked Immunosorbent Assay (ELISA)

The cells were harvested and centrifuged at 1,000 rpm for 5 min. The supernatant was used to determine the contents of IL-12, IL-2, IL-10, and IL-6 secreted by DCs, and the content of IFN-*γ* produced by CTLs using their corresponding ELISA assay kits (CLOUD-CLONE CORP., Wuhan, China) according to the manufacturer's instructions.

### 2.15. Induction of CTLs In Vitro and Sorting of CD28+ and CD8+ CTLs

The mature DCs in the control and experimental groups on the 7th day were supplemented with 10 *μ*g/mL mitomycin for 1 hr, and then cocultured with the aforementioned nonadherent cells (DC:*T* = 1 : 5). During this coculture process, 500 IU/mL IL-2 was added to the cells. The cells were cocultured for 21 days, and the CTL supernatant was obtained. Then, the CD8-PE antibody (BioLegend), CD28-FITC antibody (BioLegend), and their corresponding isotype control antibody were added to the CTL suspension and incubated in the dark for 30 min at 4°C. The cells were washed with PBS, and the cellular suspension was sorted for CD28+ and CD8+ CTLs using a FACSAria Cell Sorter (Sony, Japan).

### 2.16. Statistical Analysis

All experiments were performed independently three times, and data were expressed as the mean ± standard deviation (SD). SPSS 23.0 was used for statistical analysis, and GraphPad Prism 8.0 was employed to draw figures. Student's *t*-test was utilized for comparison between two groups. One-way analysis of variance (ANOVA) followed by post hoc Tukey's test was used for comparison involving more than two groups. A value of *P* less than 0.05 was considered as statistically significant.

## 3. Results

### 3.1. Correlation Analysis of GPC3 and Immunotherapy-Related Factors in LUSC

The 501 LUSC samples were divided into two response types nonresponder and responder, which contained 273 and 228 samples, respectively. Compared with the nonresponder samples, the *GPC3* expression was significantly lower in the responder samples (*P*=0.00099, Figure S1). Additionally, *GPC3* expression was significantly correlated with TIDE score, IFN-*γ*, MSI, and Merck18, among which the expression level of *GPC3* gene had significantly positive correlation with TIDE score (Cro. = 0.137, *P* < 0.001) and MSI (Cro. = 0.089, *P* < 0.05), whereas the expression level of *GPC3* gene had significantly negative correlation with IFN-*γ* (Cro. = −0.244, *P* < 0.001) and Merck18 (Cro. = −0.199, *P* < 0.001) (Figure S1). After analyzing the correlation between GPC3 expression and the expression of immune checkpoint genes, it was found that GPC3 expression was significantly negatively correlated with nine immune checkpoint genes, including *CD40* (Cro. = −0.238, *P* < 0.001), *CD70* (Cro. = −0.414, *P* < 0.001), *CD86* (Cro. = −0.234, *P* < 0.001), *CTLA4* (Cro. = −0.235, *P* < 0.001), *HAVCR2* (Cro. = −0.198, *P* < 0.001),*ICOS* (Cro. = −0.193, *P* < 0.001), *IDO1* (Cro. = −0.185, *P* < 0.001), *PDCD1* (Cro. = −0.208, *P* < 0.001), and *TIGIT* (Cro. = −0.147, *P* < 0.001) (Figure S1). These results indicated that GPC3 was closely related to immune response for LUSC and may become a reasonable immunotherapeutic target for LUSC.

### 3.2. GPC3 Expression in LUSC Cells and Cell Transfection Efficiency

**Figure 1:**
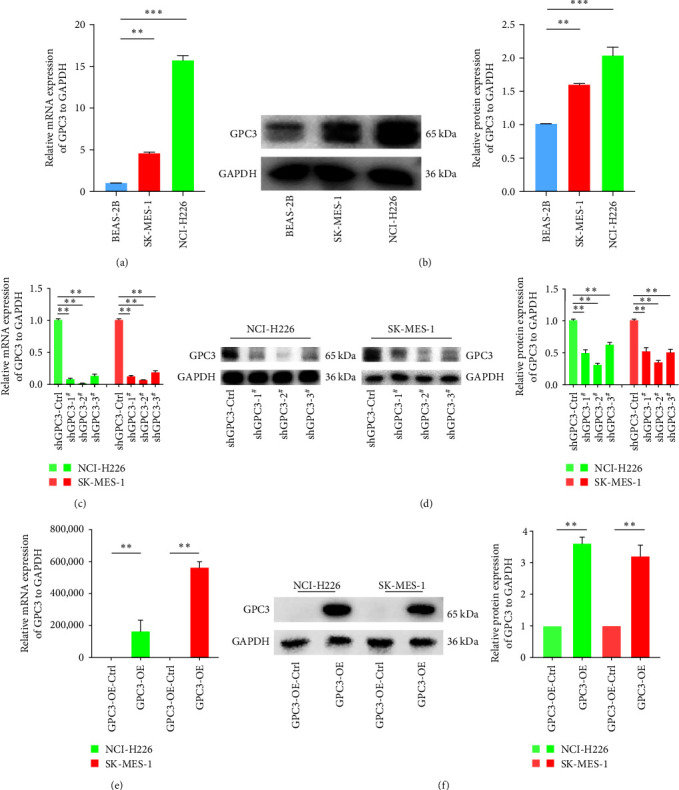
GPC3 expression in lung squamous cell carcinoma (LUSC) cell lines and cell transfection efficiency after transfection with shRNA GPC3 vectors and GPC3 overexpression vector. (a) Relative mRNA expression of GPC3 in LUSC cell lines using real-time quantitative PCR (RT-qPCR). (b) Relative protein expression of GPC3 in LUSC cell lines using western blotting. (c) Relative mRNA expression of GPC3 in cells with different treatments using RT-qPCR after transfection with shRNA GPC3 vectors. (d) Relative protein expression of GPC3 in cells with different treatments after transfection with shRNA GPC3 vectors according to western blotting. (e) Relative mRNA expression of GPC3 in cells with different treatments after transfection with overexpressed GPC3 vector according to RT-qPCR. (f) Relative protein expression of GPC3 in the cells with different treatments after transfection with overexpressed GPC3 vector according to western blotting.  ^*∗∗*^*P* < 0.01;  ^*∗∗∗*^*P* < 0.001.

The expression level of GPC3 in LUSC cells (NCI-H226 and SK-MES-1) and human normal alveolar epithelial cells (BEAS-2B) was determined by RT-qPCR and western blotting. The relative mRNA and protein expression of GPC3 in the NCI-H226 cells and SK-MES-1 cells significantly increased compared with BEAS-2B cells (*P* < 0.01, Figures 1(a) and 1(b)). This indicated that GPC3 upregulation may be involved in the occurrence and development of LUSC.

Further, shGPC3/oeGPC3 LUSC stable transfection cell lines were established to explore the specific roles of GPC3 in LUSC, with RT-qPCR and western blotting employed to determine cell transfection efficiency. The mRNA and protein expression levels of GPC3 were significantly lower than those in cells treated with shGPC3-control after transfection of NCI-H226 cells and SK-MES-1 cells with shGP3-1, shGPC3-2, and shGPC3-3 (*P* < 0.01); the effects of shGPC3-2 in LUSC cells were better (Figures 1(c) and 1(d)). These results indicated that LUSC cells transfected with shGPC3-2 could be chosen for the following experiments. The oeGPC3-NCI-H226/SK-MES-1 stable transfection cell lines were also constructed. The mRNA and protein expression of GPC3 in oeGPC3-NCI-H226/SK-MES-1 cells were upregulated compared with the GPC3-OE-control cells (*P* < 0.05, Figures 1(e) and 1(f)). These results suggested that the NCI-H226/SK-MES-1 stable transfection cells with GPC3 knockdown and overexpression were successfully built and could be used for subsequent study.

### 3.3. The Effects of GPC3 on the Proliferation, Apoptosis, and Cycle of LUSC Cells

**Figure 2:**
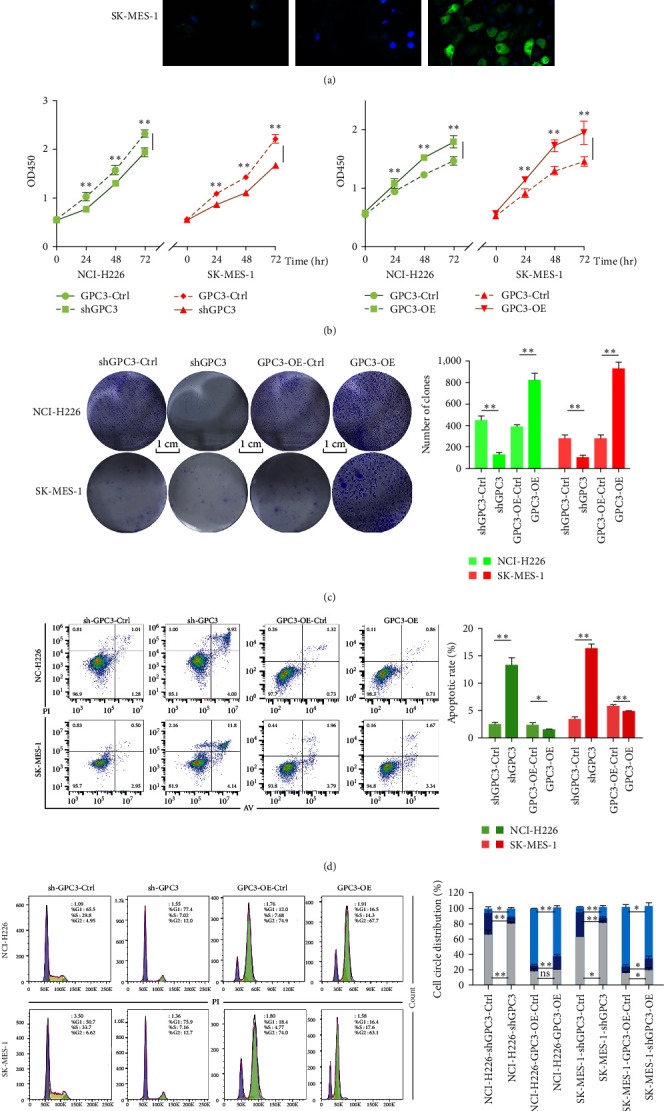
The effects of GPC3 on the proliferation, apoptosis, and cell cycle of LUSC cell lines. (a) Immunofluorescence was used to detect the expression level of GPC3 in the LUSC cell lines transfected with shRNAGPC3 vector and overexpressed GPC3 vector (400×). The green fluorescence was GPC3, and the blue fluorescence was the nucleus. (b) Cell proliferation in the LUSC cell lines transfected with shRNAGPC3 vector (left) and overexpressed GPC3 vector (right) detected by Cell Counting Kit-8 after culturing for 24, 48, and 72 hr. (c) The proliferation ability in the LUSC cell lines with different treatments using the cell clone formation method. (d) The Annexin V-FITC/PI double staining assay kit was utilized to examine the cell apoptosis of the LUSC cell lines with different treatments. (e) Cell cycle of the LUSC cell lines with different treatments detected using the PI single dyeing method. ns, no significant difference.  ^*∗*^*P* < 0.05;  ^*∗∗*^*P* < 0.01.

GPC3 was expressed in NCI-H226 and SK-MES-1 cells and was located on cytomembrane according to IF (Figure 2(a)). GPC3 was downregulated or not expressed in shGPC3-NCI-H226/SK-MES-1 cells, while the fluorescence intensity of GPC3 was enhanced compared to the control cells in the oeGPC3-NCI-H226/SK-MES-1 cells, and GPC3 expression in the cytoplasm was increased (Figure 2a).

GPC3 knockdown significantly decreased the cell viability of LUSC cells compared with the control cells after culturing for 24, 48, and 72 hr (*P* < 0.01), whereas GPC3 overexpression markedly enhanced LUSC viability of cells (*P* < 0.01, Figure 2(b)). Moreover, LUSC cell viability gradually increased with the longer culture time (Figure 2(b)). Cell clone formation results showed that the number of clones in LUSC cells with GPC3 knockdown was significantly reduced compared with the shGPC3-control-induced LUSC cells (*P* < 0.01); however, GPC3 overexpression elevated the number of clones in LUSC cells (*P* < 0.01, Figure 2(c)). These results implied that GPC3 knockdown could suppress the proliferation of LUSC cells, while GPC3 overexpression could promote their proliferation.

The total apoptosis rates in the shGPC3-control/shGPC3 groups and in the GPC3-OE-control/GPC3-OE groups in NCI-H226 cells were 2.52 ± 0.28%/13.38 ± 0.77% and 2.41 ± 0.84%/1.56 ± 0.23%, respectively (Figure 2(d)). The total apoptosis rates in the shGPC3-control/shGPC3 groups and in the GPC3-OE-control/GPC3-OE groups in SK-MES-1 cells were 3.53 ± 0.31%/16.44 ± 0.45% and 5.91 ± 0.98%/4.94 ± 0.09%, respectively (Figure 2(d)). These results manifested that GPC3 knockdown significantly induced the apoptosis of LUSC cells compared with the control cells (*P* < 0.01), whereas GPC3 overexpression inhibited cell apoptosis (*P* < 0.05). Furthermore, GPC3 silencing significantly increased G1 phase (*P* < 0.05), significantly shortened S phase (*P* < 0.01), and significantly increased G2/M phase (*P* < 0.05) compared to control cells (NCI-H226 or SK-MES-1 cells) (Figure 2(e)).The cells in the G1 phase also increased in GPC3 overexpressed cells (*P* > 0.05 in NCI-H226, while *P* < 0.05 in SK-MES-1), whereas the action of GPC3 overexpression in the S and G2/M phases of LUSC cells was opposite to that of GPC3 knockdown (Figure 2(e)). These results indicated that GPC3 could affect the growth of LUSC cells by regulating cell cycle (S and G/M2 phases).

### 3.4. The Effects of GPC3 on the Migration and Invasion of LUSC Cells

**Figure 3:**
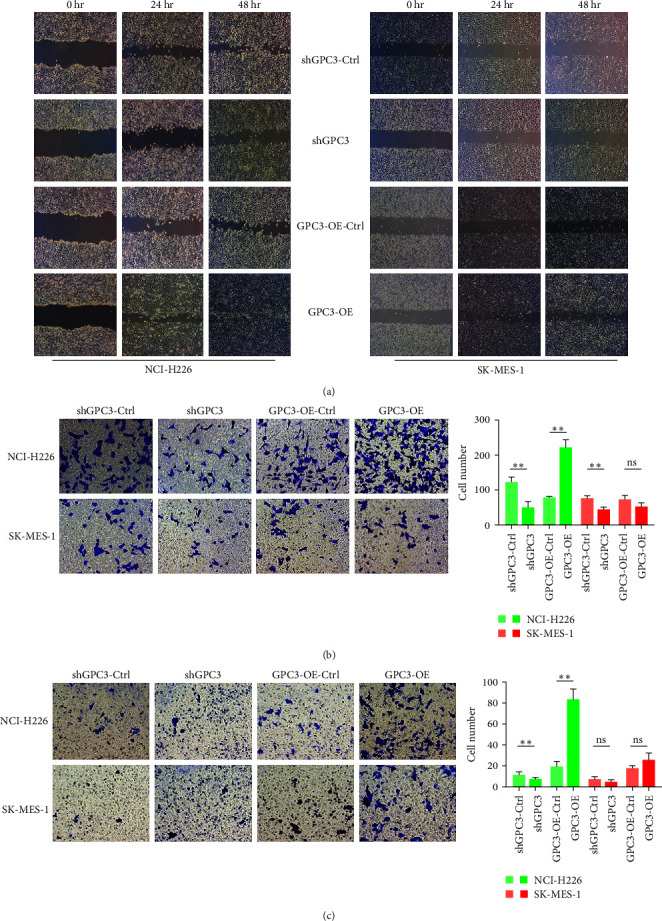
The effects of GPC3 on the migration and invasion of LUSC cell lines with different treatments. (a) The scratch test was used to determine the migration of the LUSC cells with different treatments after culturing for 24 and 48 hr. (b) The Transwell assay was also employed to test the cell migration of the LUSC cell lines with different treatments. (c) Cell invasion of LUSC cell lines with different treatments using the Transwell assay.  ^*∗∗*^*P* < 0.01; ns, no significant difference.

The scratch test showed that GPC3 silencing lowered the migration of NCI-H266 cells after 24 and 4 hr of culture, while GPC3 overexpression enhanced their migration (Figure 3(a)). However, no significant migration changes were observed in the control, GPC3 silencing, or overexpressed cells in the SK-MES-1 cells. The adherent ability of SK-MES-1 cells gradually decreased with longer observation time (Figure 3(a)). Transwell assays showed that the cell number in the shGPC3-NCI-H226 group and shGPC3-SK-MES-1 group significantly decreased compared with the control cells (*P* < 0.01). Meanwhile, the cell number in the GPC3-OE-NCI-H226 group significantly increased (*P* < 0.01), and there was no significant difference in cell number between the GPC3-OE-control-SK-MES-1 and GPC3-OE-SK-MES-1 groups (*P* > 0.05, Figure 3(b)). Subsequently, the Matrigel Transwell assay was used to determine the invasion of LUSC cells. GPC3 silencing and overexpression significantly reduced and increased the cell number (*P* < 0.01) compared to the corresponding NCI-H266 control cells, respectively (Figure 3(c)). However, no significant differences in the cell number were found between the shGPC3-control cells and GPC3 knocked-down cells in the SK-MES-1 cells and between the GPC3-OE-control cells and GPC3 overexpressed cells (*P* > 0.05, Figure 3(c)). Taken together, GPC3 knockdown inhibited migration and invasion of LUSC cells, whereas GPC3 overexpression had the opposite effects. The effects of GPC3 on NCI-H226 cells were more significant than that of SK-MES-1 cells.

### 3.5. The Effects of GPC3 on the Expression of Cell Cycle-Related Proteins and the PI3K/Akt Signaling Pathway in LUSC Cells

**Figure 4:**
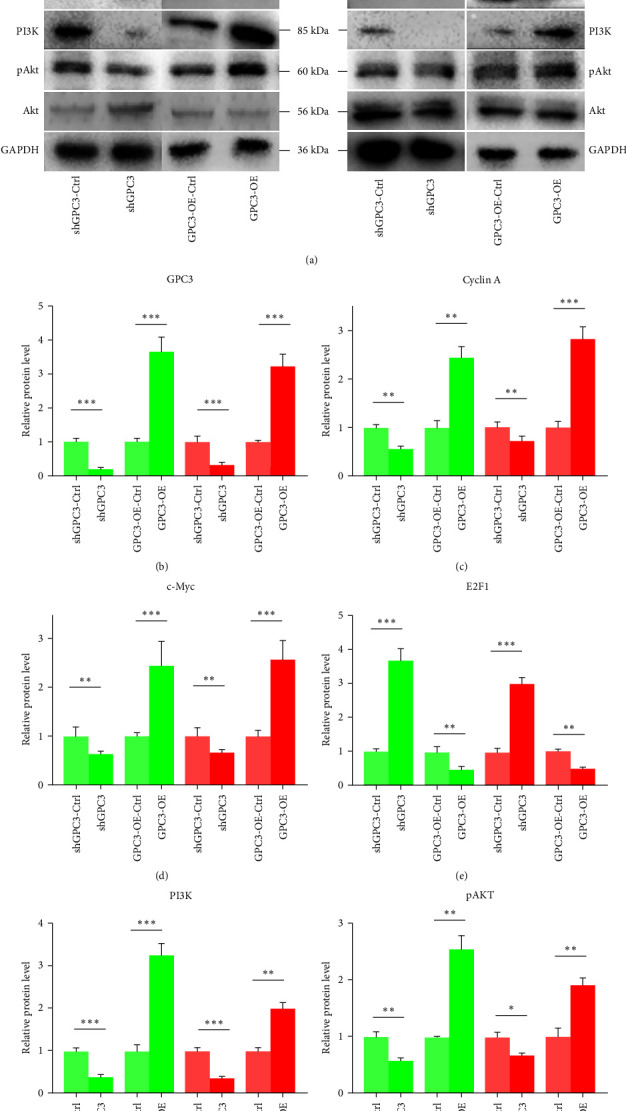
The effects of GPC3 on the expression of cell cycle-related proteins and PI3K/AKT signaling pathways in LUSC cells. (a) Representative images of the related protein bands. (b–g) Protein expression level of GPC3, cyclin A, c-Myc, E2F1, PI3K, and pAKT/AKT, respectively.  ^*∗*^*P* < 0.05;  ^*∗∗*^*P* < 0.01;  ^*∗∗∗*^*P* < 0.001.

Protein expression levels of GPC3, cell cycle-related proteins (cyclin A, c-Myc, and E2F1), and PI3K/AKT signaling pathway-associated key proteins (PI3K, pAKT/AKT) were detected using western blotting to further investigate the molecular mechanisms of GPC3 in LUSC cells. GPC3 protein expression was significantly downregulated in the shGPC3 groups than that in the control groups (*P* < 0.05); meanwhile, it was upregulated in the GPC3-OE groups (*P* < 0.05, Figures 4(a) and 4(b)). The expression levels of cyclin A and c-Myc cell cycle-related proteins were significantly reduced in the shGPC3 groups than that in the control groups (*P* < 0.05) and were markedly increased in the GPC3-OE groups (*P* < 0.05, Figures 4(a), 4(c), and 4(d)). However, E2F1 protein expression in the different groups was opposite to that of cyclin A and c-Myc (Figures 4(a) and 4(e)). GPC3 knockdown and overexpression significantly decreased and increased PI3K and pAKT/AKT levels compared to the control groups (*P* < 0.05), respectively (Figures 4(a), 4(f), and 4(g)).

### 3.6. HLA-A2 Expression in LUSC Cells and Peripheral Blood of LUSC Patients

**Figure 5:**
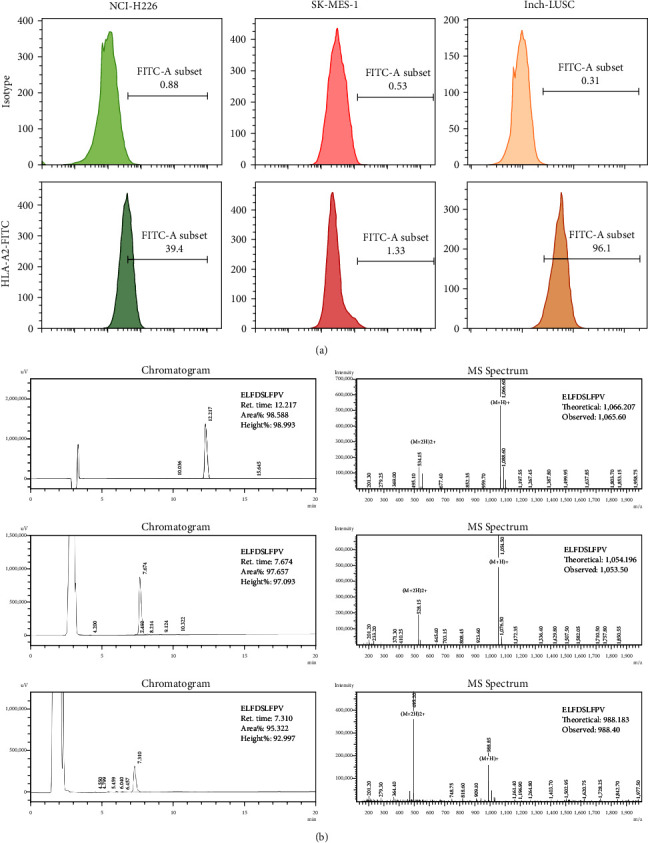
HLA-A2 expression in LUSC cells and peripheral blood of LUSC patients and identification of HLA-A2-restricted GPC3 antigenic peptides. (a) Expression of HLA-A2 in NCI-H226 cells, SK-MES-1 cells, and the peripheral blood of LUSC patients using flow cytometry. (b) Identification of HLA-A2-restricted GPC3 antigenic peptides using high-performance liquid chromatography (left) and mass spectrum (right).

There was insignificant expression of HLA-A2 in human NCI-H226 and SK-MES-1 LUSC cell lines according to flow cytometry results (Figure 5(a)). This indicated that NCI-H226 and SK-MES-1 cells did not belong to HLA-A2 positive cells.

Peripheral blood samples were collected from LUSC patients in Liaoning Cancer Hospital, and HLA-A2 expression was detected. Six cases with high HLA-A2 expression were randomly selected (Figure 5(a)). Then, the tumor tissues of the six cases were obtained for primary LUSC cell culture, and labeled as lnch-LUSC-1, lnch-LUSC-2, lnch-LUSC-3, lnch-LUSC-4, lnch-LUSC-5, and lnch-LUSC-6.

### 3.7. Synthesis and Identification of HLA-A2-Restricted GPC3 Antigenic Peptides

The full-length GPC3 amino acid sequence (Isoform1, identifier: P51654-1) was obtained from the Uniprot database (https://www.uniprot.org/uniprot/P51654), and four online databases were used to predict the GPC3 protein epitopes. Ten sequences were selected by integrating the different evaluation methods of SYFPEITHI, NetMHC 4.0, IEDB, and Propred-1 databases (Table 1). Three sequences (169 ELFDSLFPV, 522 FLAELAYDL, and 102 FLIIQNAAV) were chosen to synthesize the HLA-A2-restricted GPC3 antigenic peptides after combining the scoring indexes of the different four websites; HPLC was then used to detect the purity of the synthetic peptides. The retention time of the main peak of the target peptides ELFDSLFPV, FLAELAYDL, and FLIIQNAAV was 12.217, 7.674, and 7.310 min, respectively, and the area of the main peak accounted for 98.588%, 97.657%, and 95.322%, respectively (Figure 5(b)). Meanwhile, the actual molecular weight of the target peptides ELFDSLFPV, FLAELAYDL, and FLIIQNAAV detected by mass spectrometry was 1,065.6, 1,053.5, and 9,88.4 (Figure 5(b)). This correlated with the theoretical results (1,066.207 for ELFDSLFPV, 1,054.196 for FLAELAYDL, and 988.183 for FLIIQNAAV). These results indicated that the HLA-A2-restricted GPC3 antigenic peptides were successfully synthesized and could be used for further analysis and experiments.

### 3.8. Culture and Phenotype Identification of DCs In Vitro

**Figure 6:**
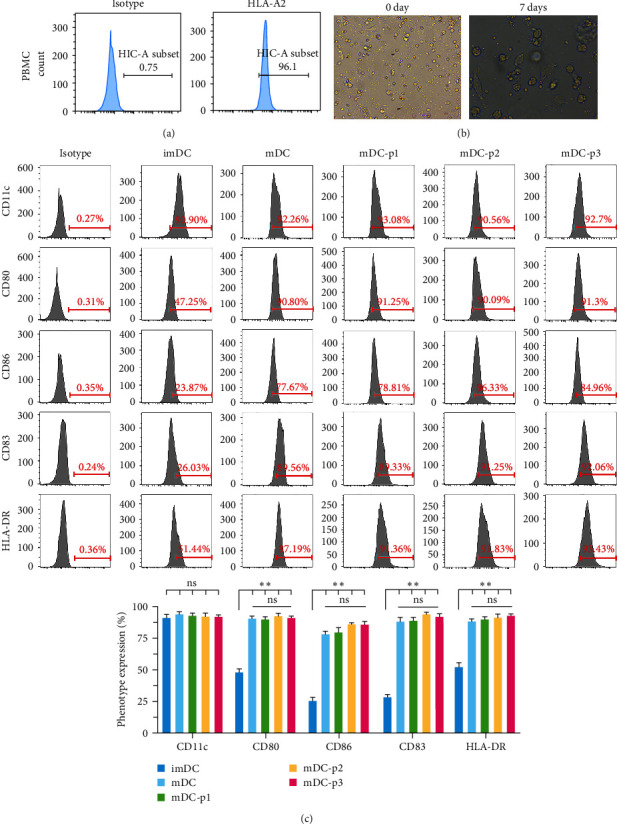
Culture and phenotype identification of dendritic cells (DCs) *in vitro*. (a) A flow cytometry was used to determine the expression of HLA-A2 in DCs after induction. (b) Morphological observation of DCs cultured *in vitro* at a magnification of 400×. (c) Molecular phenotypes of immature DCs and mature DCs detected by flow cytometry.  ^*∗∗*^*P* < 0.01; ns, no significant difference.

Flow cytometry was used to detect HLA subtypes in the peripheral blood of healthy volunteers. The peripheral blood samples of HLA-A2 subtype volunteers were selected to extract peripheral blood mononuclear cells (PBMC), and DCs were induced and cultured *in vitro* (Figure 6(a)). The cell grouping was shown as follows: imDC (immature DCs), mDC (mature DCs induced by TNF-*α*), mDC-p1 (TNF-*α*-induced mature DCs loaded with GPC3_169-178_ ELFDSLFPV antigenic peptides), mDC-p2 (TNF-*α*-induced mature DCs loaded with GPC3_522-530_ FLAELAYDL antigenic peptides), and mDC-p3 (TNF-*α*-induced mature DCs loaded with GPC3_102-110_ FLIIQNAAV antigenic peptides).

The DCs showed adherent growth, round or oval shapes, small cell volume, and smooth cell surfaces during culture at 0 day. The cells grew in suspension after 7 days, the cell volume increased, and typical dendritic pseudopodia appeared on the cell surface (Figure 6(b)). This is in line with the morphological characteristics of mature DCs.

The expression of CD11c, CD80, CD86, CD83, and HLA-DR was detected by flow cytometry to determine the proportion of DCs in the cultured cells and the phenotype of mature DCs. The expression of CD11c, CD80, CD86, CD83, and HLA-DR in the imDC group was 91.47 ± 2.24%, 48.35 ± 1.73%, 25.6 ± 2.76%, 28.30 ± 1.97%, and 52.24 ± 3.44%, respectively (Figure 6(c)). This phenotype represents imDCs. The expression of CD80, CD86, CD83, and HLA-DR in the mDC group was significantly upregulated compared to the imDC group after TNF-*α* induction (*P* < 0.01); meanwhile, no significant difference was found in CD11c expression (*P* > 0.05, Figure 6(c)). Moreover, there were no significant differences in the CD11c, CD80, CD86, CD83, and HLA-DR expression among the mDC, mDC-p1, mDC-p2, and mDC-p3 groups (*P* > 0.05, Figure 6(c)). This suggests that peptide loading and DC maturity were not closely correlated.

### 3.9. Secretion Levels of IL-12, IL-2, IL-6, and IL-10 in Different DCs

**Figure 7:**
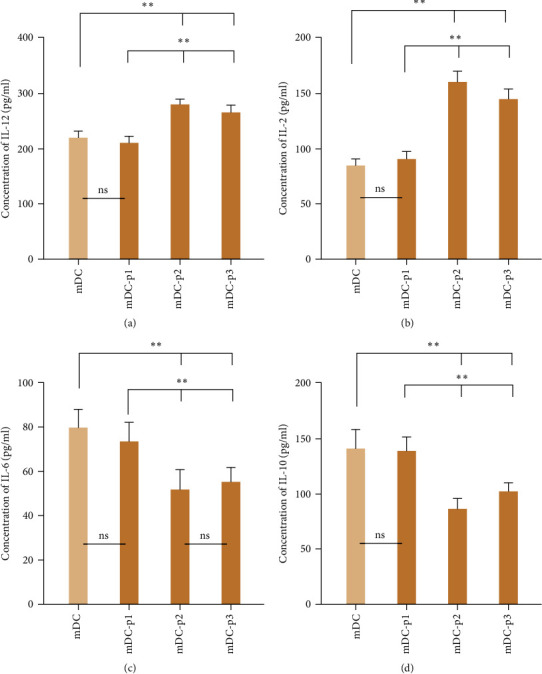
Secretion levels of IL-12, IL-2, IL-6, and IL-10 in different DCs using an enzyme-linked immunosorbent assay. (a) IL-12 concentration in the different mature DC cells. (b) IL-2 concentration in the different mature DC cells. (c) IL-6 concentration in the different mature DC cells. (d) IL-10 concentration in the different mature DC cells.  ^*∗∗*^*P* < 0.01; ns, no significant difference.

The secretion levels of IL-12, IL-2, IL-6, and IL-10 in the mDC, mDC-p1, mDC-p2, and mDC-p3 groups were measured by ELISA. The concentrations of IL-12 in the mDC, mDC-p1, mDC-p2, and mDC-p3 groups were 221.02 ± 10.89, 211.21 ± 11.41, 281.89 ± 7.4, and 265.97 ± 12.46 pg/mL, respectively (Figure 7(a)). The trend of IL-2 concentrations secreted by different DCs was similar with that of the IL-12 concentrations (Figure 7(b)). IL-6 concentrations in the mDC-p2 and mDC-p3 groups significantly declined compared with the mDC group (*P* < 0.05); meanwhile, IL-6 concentrations were not significantly different between the mDC and mDC-p1 groups and between the mDC-p2 and mDC-p3 groups (*P* > 0.05, Figure 7(c)). The concentration of IL-10 in the mDC-p2 group (87.33 ± 8.40 pg/mL) was the lowest, followed by mDC-p3 (102.13 ± 7.85 pg/mL), mDC-p1 (138.91 ± 12.64 pg/mL), and mDC (140.98 ± 16.94 pg/mL) (Figure 7(d)).

### 3.10. Optimum Killing Ratio of CTLs and Cytotoxicity Analysis in LUSC Cells

**Figure 8:**
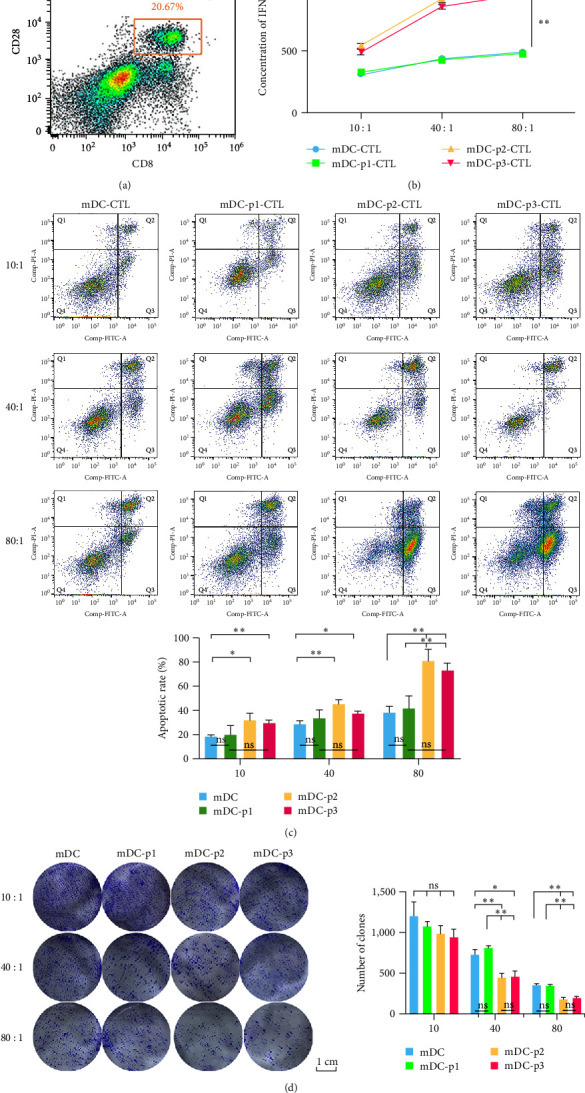
Optimum killing ratio of cytotoxic T lymphocyte (CTL) and cytotoxicity analysis in LUSC cells. (a) CD8+ and CD28+ CTL were sorted by flow cytometry. (b) IFN-*γ* concentration secreted by CTL with different multiplicity of infection. (c) Cell apoptosis induced by CTL killing of LUSC cells with different multiplicity of infection detected by flow cytometry. (d) The ability of CTL to kill target cells under different multiplicity of infection determined by the clone formation method.  ^*∗*^*P* < 0.05;  ^*∗∗*^*P* < 0.01; ns, no significant difference.

DCs modified with HLA-A2-restricted GPC3 antigenic peptides were cocultured with T cells to generate specific CTLs, and then CTLs with CD28+ and CD8+ phenotypes were sorted by flow cytometry. The CTLs after sorting were cocultured with target cells, and the sorting results are shown in Figure 8(a). Then, ELISA was utilized to measure IFN-*γ* levels secreted by nonpeptide-loaded (mDC-CTL) and peptide-loaded (mDC-p1-CTL, mDC-p2-CTL, and mDC-p3-CTL) groups at different multiplicity of infection (10 : 1, 40 : 1, and 80 : 1) *in vitro*. IFN-*γ* levels produced by DC-induced CTLs increased with higher ratios of multiplicity of infection, and the IFN-*γ* level in each group was the highest when the multiplicity of infection was 80 : 1 (Figure 8(b)). The IFN-*γ* level was higher in the mDC-p2-CTL and mDC-p3-CTL groups than that in the mDC-CTL group (*P* < 0.01), and there was no significant difference in the IFN-*γ* level between mDC-CTL and mDC-p1-CTL groups (*P* > 0.05, Figure 8(b)). These findings suggested that the three synthetic peptide-modified DCs and DCs could induce T cells to activate into CTLs, and the IFN-*γ* level secreted by mDC-p2-CTL was the highest.

Further, cell apoptosis and clone formation were determined to study the killing effects of different CTLs on LUSC cells. The total apoptosis rate increased with the increased ratio of multiplicity of infection (Figure 8(c)). The total apoptosis rate in the mDC-p2-CTL and mDC-p3-CTL groups significantly increased compared with the mDC-CTL and mDC-p1-CTL groups (*P* < 0.05, Figure 8(c)). Cell clone formation assays showed no significant difference in the number of clones among the groups at 10 : 1 multiplicity of infection (*P* > 0.05, Figure 8(d)). The number of clones in the mDC-p2-CTL and mDC-p3-CTL groups was lower than that in the mDC-CTL and mDC-p1-CTL groups at 40 : 1 and 80 : 1 multiplicity of infection (*P* < 0.05, Figure 8(d)). These results indicated that HLA-A2-restricted GPC3 antigenic peptide-loaded DCs induce CTL production *in vitro* and have targeted killing of LUSC cells. Furthermore, mDC-p2-CTL and mDC-p3-CTL have a stronger targeted killing ability at 80 : 1 multiplicity of infection.

## 4. Discussion

LUSC is a subtype of NSCLC that accounts for ∼40% of all lung cancers. It is associated with poorer clinical prognosis and lacks available targeted agents compared with LUAD [35, 36]. GPC3 was upregulated in LUSC and was involved in the regulation of cell proliferation, migration, invasion, and other biological functions by cascading different signaling pathways in cells, thus playing an oncogene role in the occurrence and development of LUSC [37]. Our study verified that GPC3 was significantly upregulated in LUSC cell lines compared with normal cells. Further, LUSC stable transfection cells with GPC3 knockdown and overexpression showed that GPC3 overexpression promoted the proliferation, migration, and invasion of LUSC cells, inhibited their apoptosis, increased the cells in S phase, and reduced the cells in G2/M phase; meanwhile, GPC3 knockdown had the opposite effects. HCC patients with higher GPC3 levels showed poor differentiation and higher proliferation levels, and GPC3 promoted the proliferation of HCC cell lines through the Hedgehog signaling pathway, thus regulating HCC progression [38]. LncRNA GPC3-AS1 and its nearby gene GPC3 are significantly upregulated in cervical cancer (CC) cells compared with normal cells; GPC3-AS1 and GPC3 synergistically enhance the proliferation and migration of CC cells by activating ELK1, thereby promoting CC development [39]. Cell cycle regulation plays an important role in the proliferation, metastasis, and recurrence of tumor cells [40]. 6-mercaptopurine antimetabolic drug mainly acts on S-phase cells, while plant alkaloids mainly act on M-phase cells that can effectively inhibit the activity and proliferation of cancer cells [41, 42]. We speculate that GPC3 silencing may repress the growth and invasion of LUSC cells through regulating cell cycle (S and G2/M phases).

Cell cycle regulation is coordinated by a complex network of interactions between proteins, enzymes, cytokines, and cell cycle signaling pathways, and is essential for cell proliferation, growth, and repair [40]. Cell cycle-related proteins were determined to further explore the molecular mechanisms of GPC3 on the cell cycle in LUSC. GPC knockdown downregulated cyclin A, c-Myc, and PI3K, upregulated E2F1, and decreased the pAKT/AKT level, whereas overexpression had the opposite effect. Cyclin A is a central and particularly interesting cell cycle regulator that functions in the S phase and mitosis [43]. Cyclin A mRNA expression and protein products start to accumulate in late G1 phase and reach a peak in S phase [44]. DUB3 overexpression increases the endogenous cyclin A levels and drives cell cycle progression by stabilizing cyclin A, thus mediating NSCLC cell proliferation [45]. C-Myc plays an important role in tumorigenesis and its regulation of cell proliferation is attributed to its ability to transcribe many genes involved in G1/S cell-cycle progression, such as CDK4 and cdc25A [46]. c-Myc regulates endogenous GPC3 expression by directly acting on the GPC3 promoter [47]. E2F1 is another core player involved in cell cycle progression, DNA damage response, and apoptosis; therefore, E2F1 inhibition may affect different levels of tumor development by blocking cell cycle progression and impounding the metabolic flexibility of cancer cells [48]. Elevated E2F1 levels activate cell cycle progression and KIF26A expression, thereby promoting the proliferation of breast cancer cells [49]. In addition, the cell cycle can be regulated by various signaling pathways, including PI3K/AKT. The PI3/AKT signaling pathway plays vital roles in the regulation of signal transduction, cell proliferation, apoptosis, metabolism, angiogenesis, and other biological processes; it is involved in the cell cycle process of cancer cells and is a potential signaling pathway for tumor treatment [40]. 4-hydroxyderricin (4-HD) treatment inhibits HCC cell growth via upregulating apoptosis-related proteins, downregulating cell cycle-related proteins, and downregulating p-AKT and p-PI3K/PI3K; however, PI3K inhibitor (LY294002) enhances the promoting effect of 4-HD on the apoptosis and cell cycle arrest of HCC cells [50]. These reports, together with our results, suggest that GPC3 may advance the onset and progression of LUSC by regulating the cell cycle (cyclin A, c-Myc, and E2F1) and the PI3K/AKT signaling pathway.

Neoantigens are tumor-specific antigens that are not expressed in normal tissues and are considered to be one of the most desirable targets to induce strong antitumor immune responses and determine the fate of tumor patients [51]. There is a significant correlation between tumor mutational antigen burden and objective response rate of checkpoint blockade immunotherapy, and a large number of clinical trials based on mutant peptides provide a strong theoretical basis for cancer treatment [52, 53]. Therefore, the discovery of mutated epitopes derived from mutated antigens has become a key part of cancer immunotherapy. Synthetic peptides as ICIs are widely used as targeted partial and therapeutic agents to treat various diseases [54]. Low-molecular weight peptides offer several advantages compared to antibodies. For example, reduced immunogenicity, ease of manufacture, better tumor penetration, and the absence of FC-mediated side effects [55]. A patient with metastatic cholangiocarcinoma has a CD4+ T-cell epitope ERBB2IP-E805G; CD4+Th1 cells targeting mutated antigens could mediate tumor regression [56]. We believed that GPC3 may act as a tumor-related specific antigen in the immunotherapy of LUSC causing the recognition of DC that can present the recognized antigen information to the initial T cells to generate specific CTL for antitumor killing. Therefore, we successfully synthesized three kinds of HLA-A2-restricted GPC3 antigenic peptides (GPC3_169-178_ ELFDSLFPV, GPC3_522-530_ FLAELAYDL, and GPC3_102-110_ FLIIQNAAV antigenic peptides) according to the full-length amino acid sequence of GPC3.

Neoantigen-targeted vaccines induce neoantigen-specific CD4+ and CD8+ T-cell responses and alter the TME [57]. Screening appropriate tumor-associated antigens and improving the efficiency of DCs to present tumor antigens are the focus and difficulty of DCs-mediated tumor immunity [58]. DC loading by a tumor antigen is an effective way to improve antigen presentation. The primary methods of tumor antigen loading include antigen sensitization and gene modification. Tumor antigen sensitization is through contact and fusion coculture, with all antigen information of tumor cells possibly transmitted to DCs; meanwhile, gene modification is where a specific gene is transfected to DCs to change the genetic expression [58]. Therefore, we loaded GPC3_169-178_ ELFDSLFPV, GPC3_522-530_ FLAELAYDL, and GPC3_102-110_ FLIIQNAAV antigenic peptides onto mature DCs to further investigate the killing effects of these peptides on LUSC cells via sensitizing DCs.

DCs have immune activation and immune tolerance induction, which are closely related to the developmental and mature state [59]. DC precursors first differentiate into immature DCs that have strong antigen recognition and migration ability. Immature DCs migrate to lymph nodes and differentiate into mature DCs upon antigen uptake. MHC-II and costimulatory signaling molecules (such as CD80, CD86, CD83, and HLA-DR) are highly expressed on the surface of mature DCs that are presented to CD8+ T lymphocytes by cross-presentation of the MHC-I pathway and then activate T cells [60]. Activated T cells are known as effector T cells and may kill tumor cells. Our study used TNF-*α* to induce DC maturation *in vitro* and found that the expression of CD80, CD86, CD83, and HLA-DR significantly increased compared with immature DCs. There were insignificant differences in the expression of CD80, CD86, CD83, and HLA-DR mature DCs and mature DCs after loading with GPC3_169-178_ ELFDSLFPV, GPC3_522-530_ FLAELAYDL, and GPC3_102-110_ FLIIQNAAV antigenic peptides. These results indicated that mature DCs were successfully induced, and the induction of mature DCs by TNF-*α* was unaffected by peptide loading.

DCs can simultaneously secrete different cytokines in the process of DC differentiation and maturation that have the negative and positive function of regulating DCs and T cells [61]. Cytokine IL-12 could not promote T-cell proliferation alone and mediate specific antitumor cytotoxic effects in the absence of APC activation [62]. Additionally, it is necessary to add a certain dose of IL-2 to maintain the survival and growth of T cells when CTLs are induced and cultured *in vitro*. The application of high doses of IL-2 in *in vivo* experiments can induce the production of regulatory T cells (Tregs), a type of regulatory T cell with inhibitory effects *in vivo* [63]. IL-6 is a pleiotropic cytokine that is highly produced in tumor-bearing hosts and inhibits the antigen presentation ability of DCs by activating signal transducer and activator of transcription 3 (STAT3) [64]. IL-10 is an important factor that enables tumor cells to escape immune attack. It inhibits the maturation of DCs and promotes their differentiation into macrophages and suppress antigen presentation of DCs, thereby weakening their ability to activate T cells [65]. Our study showed that GPC3_522-530_ FLAELAYDL and GPC3_102-110_ FLIIQNAAV antigenic peptides significantly increase the levels of IL-12 and IL-2, whereas decreased IL-6 and IL-10 levels were secreted by mature DCs; however, GPC3_169178_ ELFDSLFPV antigenic peptide did not affect these levels.

The antitumor effect of DC vaccine is achieved by inducing T-cell activation, that is, producing CTLs. CTLs are known as CD8+ T lymphocytes and are the main effector cells in cellular immune response; they can improve the specific killing of effector cells to tumor cells through efficient antigen presentation [66]. Activation of CTLs usually requires TCR-pMHC molecule specific binding, costimulatory molecular signals (CD80/CD86, CD40/CD40L, ICOS/ICOS-L), and cytokine signals. The killing mechanisms of CTLs in specific antitumor cells include the granule exocytosis pathway, Fas/FasL pathway, and cytotoxic cytokine pathways (such as TNF-*α* and INF-*γ*) [67]. Effector CD8+ T cells in the TME can produce IL-2, IL-12, and IFN-*γ*, and cytotoxicity improvement of CD8+ T cells can promote targeted killing of tumor cells. Our study showed that GPC3_169-178_ ELFDSLFPV, GPC3_522-530_ FLAELAYDL, and GPC3_102-110_ FLIIQNAAV antigenic peptides modified DCs, and DCs activate CTLs, with GPC3_522-530_ FLAELAYDL and GPC3_102-110_ FLIIQNAAV antigenic peptides facilitating more IFN-*γ* secretion. In addition, Shimizu et al. [68] found that HSP105 peptide-specific CTLs could induce immunological effects in patients with colorectal cancer and improve their prognosis. It can be inferred that GPC3_522-530_ FLAELAYDL and GPC3_102-110_ FLIIQNAAV antigenic peptides may have stronger affinity with DCs and induce CTLs to produce stronger targeted killing ability for LUSC cells at 80 : 1 multiplicity of infection.

## 5. Conclusions

GPC3 may promote the growth, migration, and invasion of LUSC cells via regulating the cell cycle (S and G2/M phases, the expression of cyclin A, c-Myc, and E2F1) and the PI3K/AKT signaling pathway, thus accelerating the onset and progression of LUSC. Additionally, GPC3_522-530_ FLAELAYDL and GPC3_102-110_ FLIIQNAAV antigenic peptides-loaded mature DCs-induced CTLs could have stronger killing ability against LUSC cells, and the optimal multiplicity of infection could be 80 : 1. These findings lay the foundation for treating LUSC with GPC3 as a new target and provide candidate novel epitopes to develop tumor vaccines and immunotherapy for LUSC patients.

## Figures and Tables

**Table 1:** Prediction results of HLA-A2-restricted GPC3 antigenic peptide using different databases.

Rank	Sequence	At position	SYFPEITHI	NetMHC4.0	ProPred-1	IEDB
1	RLQPGLKWV	44	27	1.3	37.97	0.68
2	FLAELAYDL	522	27	0.003	33.59	0.89
3	FLIIQNAAV	102	26	0.15	32.3	0.65
4	VLLGLFSTI	319	26	0.5	27.03	0.46
5	YILGSDINV	155	24	0.2	28.52	0.67
6	VMQGCMAGV	281	24	0.2	29.57	0.17
7	LLTSMAISV	564	23	0.2	26.73	0.53
8	ELFDSLFPV	169	23	0.01	38.99	0.84
9	YILSLEELV	299	23	0.2	24.52	0.50
10	TIHDSIQYV	326	23	0.5	34.76	0.87

## Data Availability

The datasets used and/or analyzed during the current study are available from the corresponding authors on reasonable request.

## Supplementary Materials

Supplementary Materials Figure S1: correlation analysis between *GPC3* and immunotherapy-related factors in lung squamous cell carcinoma (LUSC).
