# Supplementary material for: GPC3 Promotes Lung Squamous Cell Carcinoma Progression and HLA-A2-Restricted GPC3 Antigenic Peptide-Modified Dendritic Cell-Induced Cytotoxic T Lymphocytes to Kill Lung Squamous Cell Carcinoma Cells
Source: J Immunol Res. 2023 Nov 6;2023:5532617. doi: 10.1155/2023/5532617 (PMC10643027; doi:10.1155/2023/5532617)
Supplement: Supplementary Materials — Figure S1: correlation analysis between GPC3 and immunotherapy-related factors in lung squamous cell carcinoma (LUSC). [file 5532617.f1.docx]

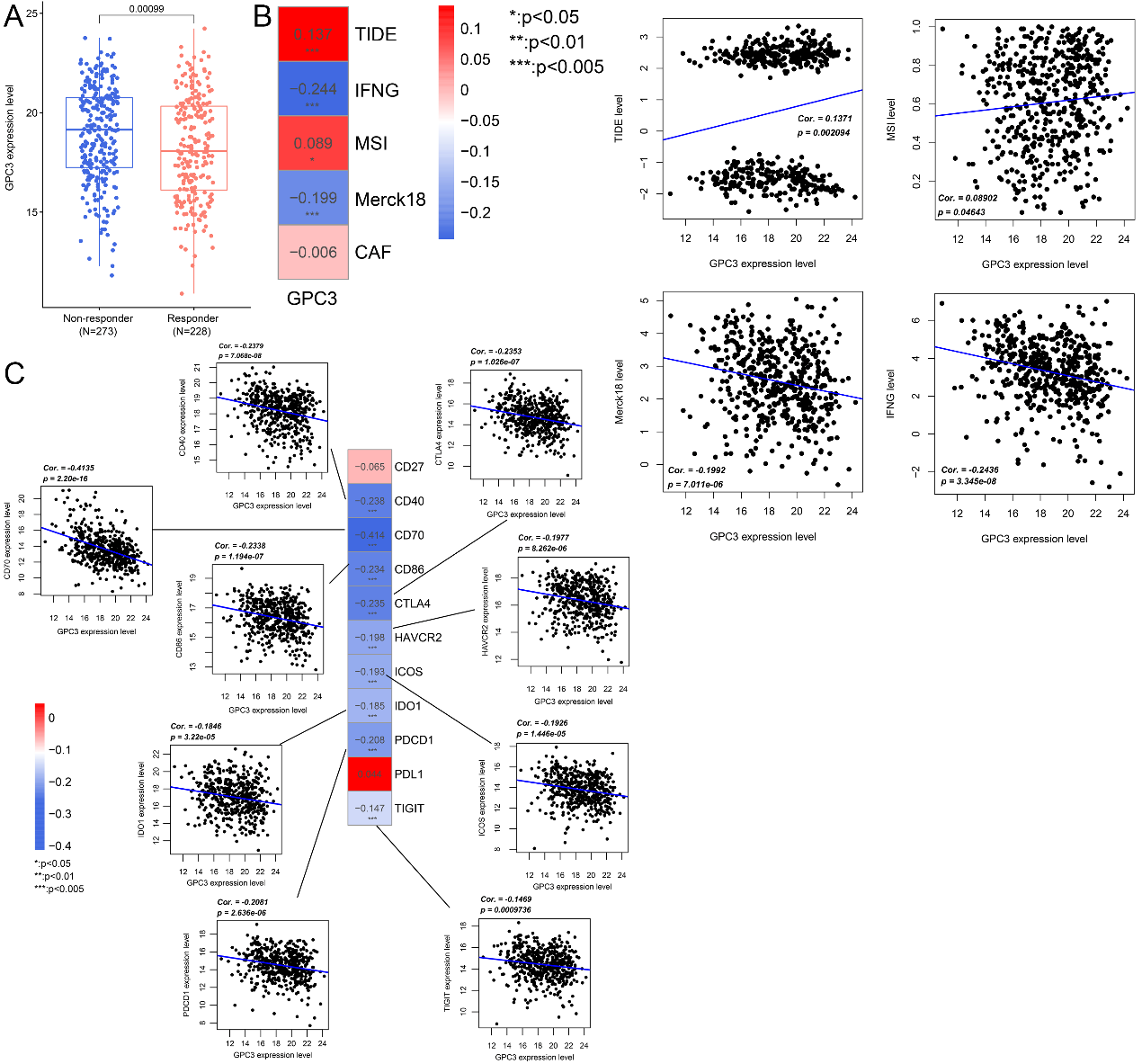


**Figure S1** Correlation analysis between *GPC3* and immunotherapy-related factors in lung squamous carcinoma (LUSC). (**A**) The GPC3 expression between in LUSC samples of immune responder and immune non-responder. (**B**) The heatmap (left) and scatter diagrams (right) of the relationship between the GPC3 expression and some indicators of immunotherapy, including TIDE score, IFNG (interferon-γ), MSI (microsatellite instability), Merck18, CAF (tumor-associated fibroblasts). (**C**) The correlation between GPC3 expression and the expression of immune checkpoint genes (*CD27*, *CD40*, *CD70*, *CD86*, *CTLA4*, *HAVCR2*, *ICOS*, *IDO1*, *PDCD1*, *PDL1*, *TIGIT*). *: *P* < 0.05, **: *P* < 0.01, ***: *P* < 0.001.
